# Endoscopic combined intrarenal surgery in the prone split-leg position versus Galdakao-modified supine Valdivia position for the management of partial staghorn calculi

**DOI:** 10.1186/s12894-022-01115-3

**Published:** 2022-10-20

**Authors:** Tamer A. Abouelgreed, Mohamed A. Abdelaal, Moamen M. Amin, Adel Elatreisy, Osama Shalkamy, Abdrabuh M. Abdrabuh, Osama M. Ghoneimy, Hamdy Aboutaleb

**Affiliations:** 1 Department of Urology, Faculty of medicine, Al-Azhar University, Cairo, Egypt; 2 Gulf medical university, Ajman, UAE; 3 Department of Urology, Faculty of medicine, Menoufia University, Shibin el Kom, Egypt

**Keywords:** Intrarenal surgery, Prone, Staghorn, Supine

## Abstract

**Objective:**

To evaluate and compare the outcome of ECIRS in the treatment of partial staghorn renal calculi in both prone split-leg positions versus GMSV positions with regard to; technical aspects, success rate, operative time, complications, safety, and effectiveness of both approaches.

**Patients and methods:**

Between October 2018 and August 2021, 66 patients with partial staghorn calculi were enrolled in this prospective comparative study. Patients were randomly divided according to a 1:1 ratio into two groups. Group A included 33 patients who were treated by (ECIRS) in the prone split-leg position, and group B included 33 patients who were treated by (ECIRS) in the Galdakao-modified supine Valdivia (GMSV) position.

**Results:**

No significant statistical difference between both groups regarding the mean age (p = 0.448), mean body mass index (BMI) (p = 0.137), mean stone burden (p = 0.435), mean operative time (p = 0.541) and the number of calyces located in branched stones (p = 0.628). The mean hospital stay was 6.71 ± 1.12 days for group A and 6.66 ± 1.10 days for group B patients (p = 0.724). The final SFR was achieved in (29)87.87% and (30)90.9% of group A & B patients, respectively (p = 0.694). No significant difference was detected between both groups in perioperative complication rates.

**Conclusion:**

ECIRS is safe and effective in treating partial staghorn calculi either in the prone split-leg position or in the Galdakao-modified supine Valdivia position, with comparable outcomes and no statistically significant difference between both positions.

## Background

Staghorn calculi are large, branched stones occupying the renal pelvis and one or more calyceal extensions. They can be complete or partial, depending on the collecting system’s occupancy level [1]. PNL (percutaneous nephrolithotomy) is recommended as the modality of choice for treating staghorn renal calculi by the EAU and AUA guidelines [2, 3]. However, when the renal calculi are complex, including large branching staghorn calculi or multiple renal stones, the clearance may require an extensive renal multichannel approach. PNL with a single-channel approach associated with numerous and repeated attempts by the nephroscope to reach each calyx could damage the calyx neck causing multiple complications such as bleeding, blood transfusion, and urinary extravasation [4]. On the other hand, PNL with a Multichannel nephroscopic approach is associated with significant renal parenchymal injury [5]. RIRS (Retrograde Intrarenal Surgery), a single modality for treating large stones, carries the risk of extravasation and the spread of infection due to high intrarenal pressure.

ECIRS (Endoscopic combined intrarenal surgery) combines PNL with RIRS in a minimally invasive method for treating complex upper urinary tract calculi. This combination has many advantages, as it can reduce the repeat of operations and decrease the number of puncture channels used to treat complex upper urinary tract stones [6, 7]. Another advantage of ECIRS is its ability to improve the stone clearance rate in a single operation [8]. The position is the cornerstone to do a perfect combination of PNL and RIRS. The most popular position is the Galdakao-modified supine Valdivia (GMSV) position. [6, 7, 9, 10]. It has the advantage of facilitating anesthesia management with no significant effect on the circulation or respiration of the patient [6].

Conversely, it has some disadvantages, including the limitation of the puncture site, especially for an upper calyceal puncture with a risk of visceral injury [11]. Many urologists are familiar with the prone position in treating renal calculi by percutaneous nephrolithotomy. Most also perform the prone split-leg position (PSL) in ECIRS, adding ample puncture space [12, 13].

Our prospective study aims to evaluate and compare the outcome of ECIRS in treating partial staghorn renal calculi in both prone split-leg positions versus GMSV positions concerning technical aspects, success rate, operative time, complications, safety, and effectiveness of both approaches.

## Patients and methods

Between October 2018 and August 2021, 80 patients who presented with partial staghorn calculi were enrolled in this prospective comparative study. The sample size was calculated utilizing the G-power software program for statistical power 80% and type II statistical error 20%. 66 Patients who complete the study were randomly (computer-generated randomization) divided into two groups according to a 1:1 ratio. Group A included 33 patients who were treated by (ECIRS) in the prone split-leg position and completed the follow up protocol. Similarly, group B included 33 patients who were treated by (ECIRS) in the Galdakao-modified supine Valdivia (GMSV) position. The Research Ethics Committee approved the study protocol at Thumbay University hospital (affiliated with Gulf Medical university). We excluded all patients with coagulation disorders, congenital anomalies in the kidney such as horseshoe kidney, ectopic pelvic kidney, previous PNL, stricture of the ureter of the target side, severe urinary tract infection, or tuberculosis, and severe cardiac and pulmonary dysfunction; similarly, we excluded any patient with concomitant ureteric calculi of the same surgical site that may affect the procedure or its time. Informed written consent was taken from all participating patients. All patients underwent preoperative evaluation, including the history, clinical examination, routine laboratory investigations including basal parameters and urine culture, and anesthesiology risk evaluation according to the American Society of Anesthesiologists (ASA scoring); we included ASA 1 and 2. Patient demographics are displayed in Table 1. All patients were assessed by an unenhanced computed tomography (CT-KUB) scan and a plain film of the abdomen. Preoperative imaging studies evaluated the stones’ location, size, and density. We defined the stone size by measuring the longest diameter of the stone on a CT scan. In the case of multiple stones, it was calculated as the sum of the longest diameter of each stone.

**Table 1:** Demographic and clinical characteristics of patients in both groups

		Group A (n = 33)	Group B (n = 33)	P value
**Mean age ± SD(yr)**		44.74 ± 12.82	42.56 ± 13.36	0.448
**Gender**	**Male (n = 39)**	20	19	0.695
	**Female (n = 27)**	13	14	
**Body mass index (kg/m** ^**2)**^		22.12 ± 3.14	23.20 ± 2.67	0.137
**Stone side**	**Right**	17	16	0.875
	**Left**	16	17	
**Stone burden (mm** ^**2**^ **)**		665.28 ± 229.74	678.29 ± 223.11	0.435
**Grade of hydronephrosis**	**None**	8	7	0.896
	**Mild**	11	11	
	**Moderate**	12	13	
	**Sever**	2	2	
**Calyceal location**	**Upper**	19	21	0.628
	**Middle**	17	14	
	**Lower**	21	23	

The stone surface area was estimated using the formula described by Tiselius and Andersson (length × width × 3.14 × 0.25) [14]. Urine bacterial culture was completed in all patients before admission, and appropriate antibiotics were selected before the operation accordingly.

There were no patients with preplaced stents in our study.

## Surgical techniques

**Fig. 1:**
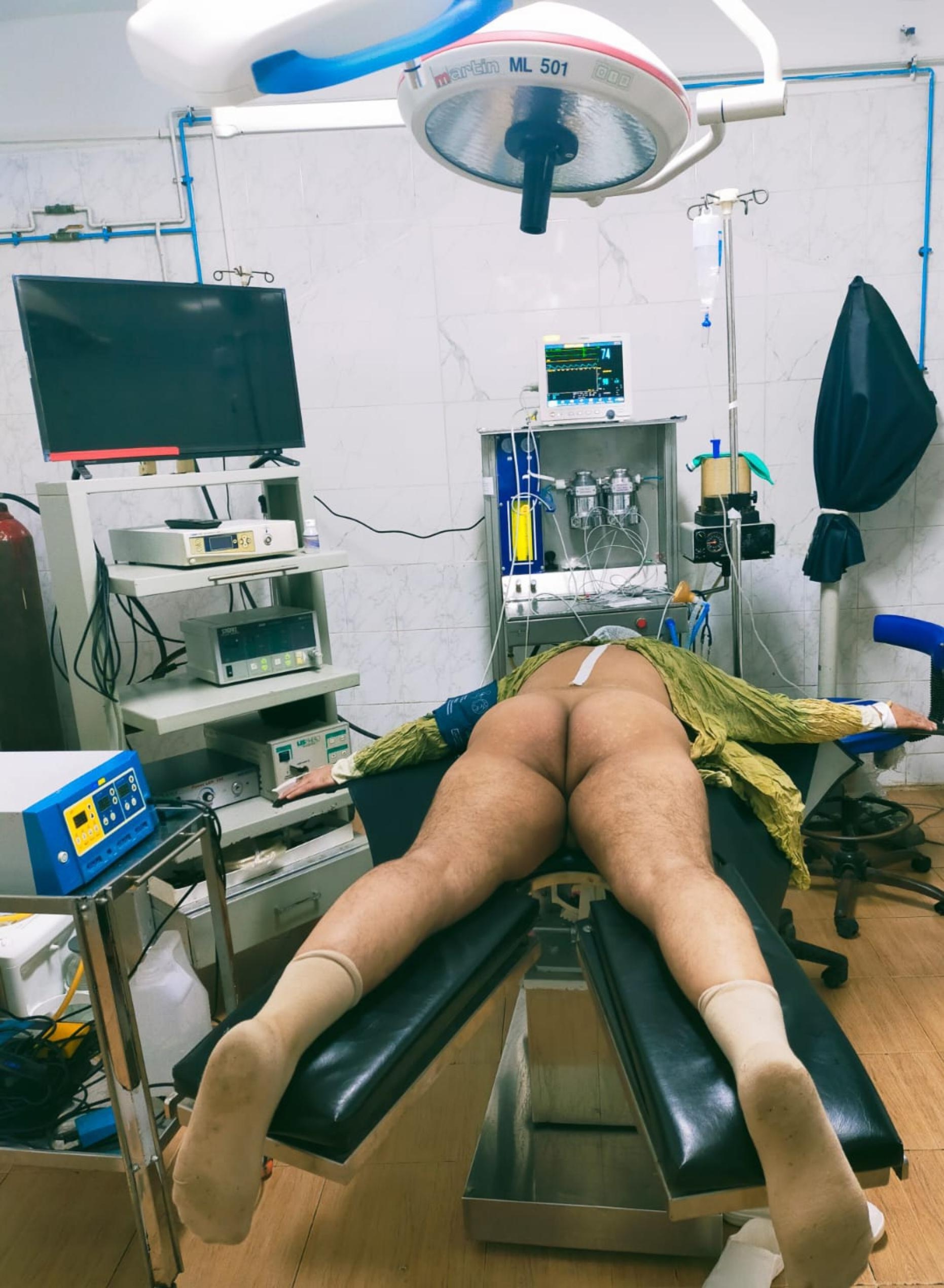
The prone split-leg position

**Fig. 2:**
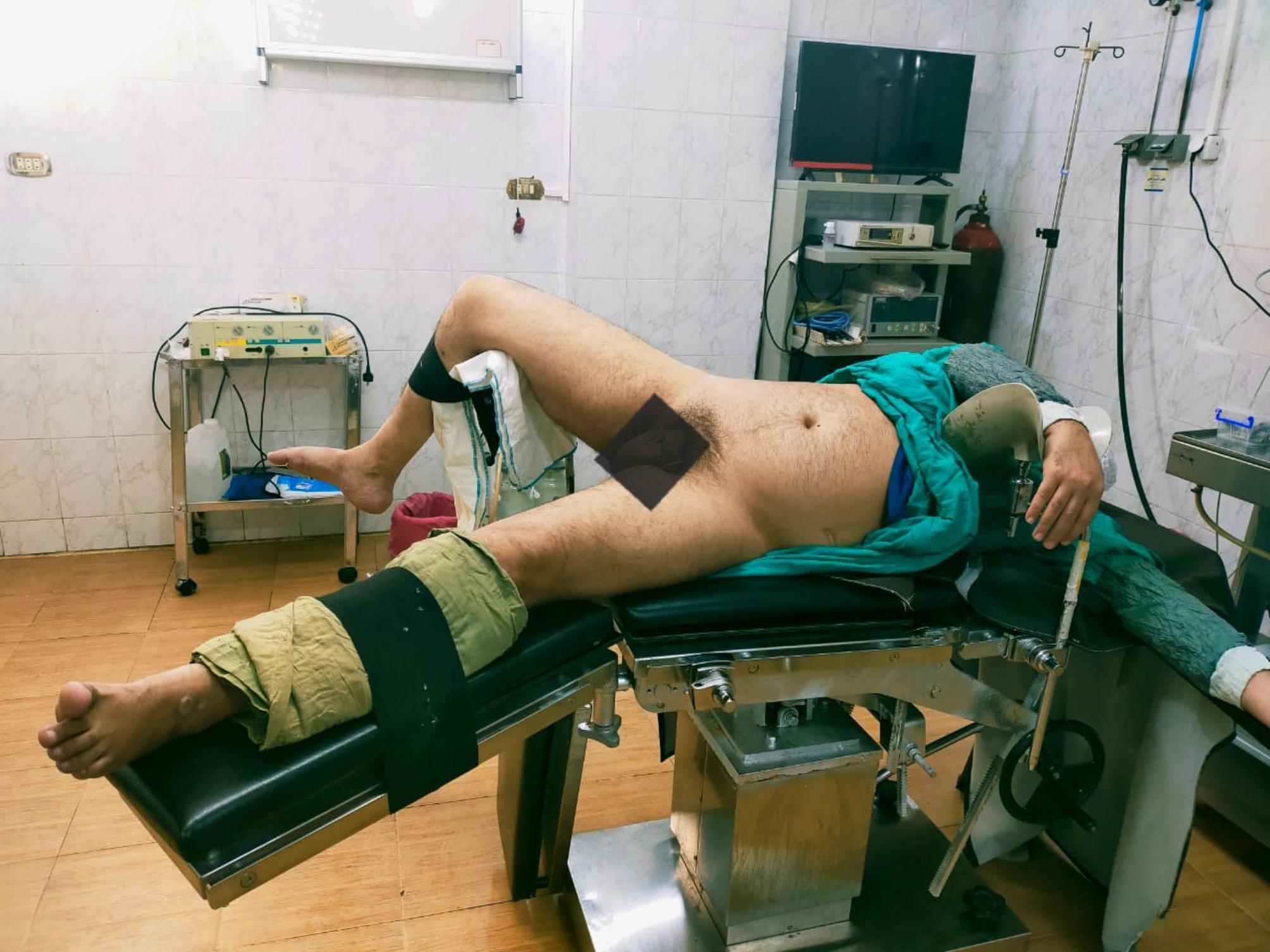
The Galdakao-modified supine Valdivia position

All procedures were performed under continuous epidural anesthesia. The patient was oriented in the prone split-leg position (group A), as shown in (Fig. 1), or the Galdakao-modified supine Valdivia (GMSV) position (group B), as shown in (Fig. 2). Flexible cystoscopy was performed to locate the ureteral orifice, easily observed with the patient in the prone position. Under fluoroscopic guidance, the ureteral orifice was cannulated with a 0.035-mm guide wire that was passed into the upper urinary tract, while a 10-F/12-F ureteral access sheath was inserted to enable frequent passage of the ureteroscope 7.5 F (Flex X-2; Karl Storz, Tuttlingen, Germany) to the renal calculi sites. Fluoroscopic guided percutaneous puncture of the lower or middle calyx was done after injection of contrast in the pelvicalyceal system, J-tip guidewire was inserted, and a safety guidewire, dilation of the tract using a plastic Amplatz dilator up to 18 F. Two urologists worked simultaneously to fragment the partial staghorn calculi. One performed RIRS using a Holmium-Yag laser with a ureteroscope through a ureteral access sheath. A single-action pumping system (SAPS; Boston Scientific, USA) was used for controlled irrigation during RIRS. A 200 Holmium-YAG laser fiber was used with RIRS to fragment the renal calculi that the basket cannot grasp due to the narrow calyceal infundibulum. The commonly used laser settings were 0.5 to 1.0 J at 5 to 10 Hz. The second urologist performed PNL using combined ultrasound and pneumatic lithotripsy (AMS, Switzerland) with vacuum suction through the 18-F mini-PNL tract (Karl Storz) [12], and smaller fragmented stones were washed through the mini-PNL tract by retrograde irrigation, or stone basket or grasping device was used to transfer calyceal stones into the renal pelvis, where the mini-PNL group can use concomitant lithotripsy and remove the fragments efficiently. At the end of the procedure, the urinary tract was stented with a 6-F Double-J ureteral stent and an 18-F nephrostomy tube. The blood loss was determined by measuring the mass of hemoglobin in the intraoperative irrigation fluid and urine. We sent for stone analysis in all patients after the operation. The nephrostomy tube was removed two days postoperatively. After removing their nephrostomy tube, patients were discharged home, provided no significant residual stone on KUB

## Post-operative evaluation

On day one, after surgery, all patients underwent laboratory investigations, including measurements of hemoglobin and hematocrit levels to be compared with those before the operation. CT-KUB was repeated 1–3 months from surgery to define the stone clearance.

## Follow-up

One month following the final procedure, all patients were assessed by CT-KUB to define the final stone-free rate (SFR). We defined the complete stone-free status with the absence of any kidney fragments or clinically insignificant residual fragments (CIRFs), including 4 mm or smaller non-symptomatic, non-obstructive, and noninfectious fragments [15]. After confirming the stone-free status, the patients were booked for flexible cystoscopy and Double-J stent removal. Complications were graded according to the modified Clavien classification [16]. All patients were reassessed with urine analysis, with serum creatinine, KUB, and KUB/ultrasound every three months for one year. CT-KUB evaluation was offered for patients with stone recurrence or increased stone size to guide the appropriate treatment modality.

## Statistical methods

We utilized SPSS (IPSS Statistics for Windows, version 28.0; IBM, Armonk, NY, USA) for data analysis. Continuous variables were summarized as the mean ± SD. Frequencies and percentages represent categorical variables. The chi-square test was used to compare categorical variables, and Student’s t-test was applied for continuous variables of the treatment groups. p < 0.05 indicated significant findings.

## Results

Sixty-six patients with partial staghorn calculi were enrolled in this study. Table 1 shows the demographics and clinical characteristics of patients and stones for each group. The mean operative time was 118.87 ± 27.12 min in group A and 121.54 ± 26.73 min in group B, with an insignificant p-value. The mean blood loss was 125.55 ± 36.70 ml and 124.78 ± 34.88 ml in groups A and B, respectively (p = 0.340) (Table 2).

**Table 2:** Operative characteristics of patients

			Group A (n = 33)	Group B (n = 33)	P value
**Operative times (minutes)**			118.87 ± 27.12	121.54 ± 26.73	0.541
**Blood loss (ml)**			125.55 ± 36.70	124.78 ± 34.88	0.340
**Complications** **of surgery**	Post-operative fever (> 38.5 C)		2	1	0.918
	Urosepsis		1	2	
	Urine leakage		2	1	
	Significant hemorrhage		1	2	
	Blood transfusion		1	1	
**Initial SFR (n)%**			(26)78.78%	(27) 81.81%	0.694
**Final SFR (n)%**			(29) 87.87%	(30) 90.9%	0.658
**Ancillary treatment**		flexible ureteroscopic lithotripsy	3	2	0.921
		MPCNL	2	2	
		ESWL	2	2	
**Hospitalization time (day)**			6.71 ± 1.12	6.66 ± 1.10	0.724

Table 2 shows the postoperative complications reported in 21.2% of the study population. The postoperative complications in group A included two transient fevers (> 38.5 C) and one urosepsis (due to E. Coli) (Modified Clavien Classification, grade 2); 2 patients displayed urinary leakage (grade 1); one patient had a post-procedural significant hemorrhage and required blood transfusion (mean blood loss was 530 ml). For group B, postoperative complications included one transient fever, two urosepsis (one due to E. coli and another due to Enterococcus faecalis), one urinary leakage, and two patients who developed significant hemorrhage (one required blood transfusion, blood loss of 565 ml). All cases of urosepsis in our study were responsive to anti-infective treatment according to urine culture results. No patient developed grade 3 complications. No significant difference was detected between both groups’ perioperative complication rates (Table 2). The mean hospital stay was 6.71 ± 1.12 days for group A and 6.66 ± 1.10 days for group B patients (p = 0.724).

The initial SFR (one week after the first stage operation) was achieved in (26)78.78% and (27)81.81% of group A & B patients, respectively (p = 0.694). Clinical significant residual stones were detected in 13 (19.6%) patients (7 of group A and 6 of group B) after a single session of ECIRS. Five patients (3 group A & 2 group B) received ancillary treatment with flexible ureteroscopic lithotripsy. Four patients (2 of each group) received additional treatment with mini-PNL, and four patients (2 of each group) received additional treatment with ESWL. The final SFR was determined three months after ancillary treatment, and the results showed that the final SFR was 87.87% in group A and 90.9% in group B (p = 0.658) (Table 2).

## Discussion

Percutaneous Nephrolithotomy is recommended in treating large renal stones exceeding two centimeters [17]. However, ECIRS, Combining PNL with RIRS in one position, is the key issue when dealing with complex renal calculi as it makes intracorporeal lithotripsy more effective, decreases intrarenal pelvic pressure, decreases the operative time required for stone clearance, and has fewer complication rate compared to PNL monotherapy [1–7].

The widely used two positions in ECIRS, GMSV, and PSL, are achieving good results nowadays. The GMSV position has little influence on cardiovascular and respiratory movement after general anesthesia, especially in obese patients, and is close to the daily physiological position of patients [18]. However, the disadvantages of this position are that the space for renal puncture is limited, and the difficulty is increased, especially in the upper calyces, which will increase the risk of visceral injury. In addition, decreased perfusion pressure is due to the influence of gravity during PNL, and the renal collecting system cannot be filled adequately. The aggregation of bubbles will affect the clarity of vision [19, 20]. While there is a broader space for the puncture in the PSL position, the forward movement of the kidney and ureter gives easy access to the ureteral sheath.

Moreover, urologists generally are more familiar with that position [12]. Posterior calyceal and proximal ureteral calculi will be collected in the renal pelvis under the effect of gravity and irrigation to be easily seen by the nephroscope. The depth of the puncture tract is shorter and can be repeated with more than one access in the prone position, which improves the safety of PNL [11]. The learning curve of ureteroscopy in the prone position is short. In addition, there was no ureteral injury caused by ureteroscopy in our study. Regarding the comparison between GMSV-ECIRS and PSL-ECIRS in SFR, Our study reported no significant difference (87.87% in PSL Vs. 90.9% in GMSV). The initial renal stone-free rate (SFR) for all patients was 80.3% (78.78% in PSL and 81.81% in GMSV), and 19.7% of all patients required secondary treatment. Our results go to a recent study by Wang et al. [21], who reported that the initial renal stone-free rate was 78.1%, and 11.5% of patients required secondary treatment. Manikandan et al. also demonstrated a similar success in managing complex renal stones, with 18% of patients requiring secondary treatment [9]. Hamamoto et al. showed nearly the same SFR as our study. He reported an 83.3% renal stone-free rate in PSL-ECIRS [12]. Most reports indicate that the renal stone-free rate of ECIRS in the GMSV position is 65.3– 87.9% [10, 22–24]. Theoretically, the position might add some difficulty in puncture during GMSV-ECIRS. Still, it will not affect the synergistic effect of combining RIRS with PNL. Staghorn stones and the number of stone branches rather than patient position were identified as independent predictors of SFR following ECIRS [8, 9]. Many studies reported a lower complication rate in GMSV-ECIRS, the restricted excessive torquing of the nephroscope because of limited manipulation area reduces the risk of intraoperative bleeding [10–12]. The supine position in GMSV facilitates the anesthesia management of cardiovascular and respiratory systems, specifically in obese patients [13, 14]. Additionally, the percutaneous tract in PSL-ECIRS is in an upward position that increases intrarenal pressure and facilitates fluid absorption [10–12]. The current study reported a 21.2% complication rate, significantly lower than that of Manikandan et al., who reported a 32.5% complication rate of ECIRS in the GMSV position [9]. Our complication rate is also lower than that of Cesare et al., who reported a 38.6% complication rate of ECIRS in the GMSV position [25]. There was no grade 3 or above complications in our study. The current study revealed no statistically significant differences between both groups regarding operative time, blood loss, complication rate, and hospitalization time (Table 2). ECIRS has apparent advantages in reducing complications. Starting by monitoring the puncture by the ureteroscope to avoid injury; manipulating the stone in parallel calyces that are away from access to the nephroscope; in addition, direct lithotripsy can be performed to avoid the risk of bleeding caused by excessive swing of the nephroscope, lowering the intrarenal pressure by using two channels to decrease the risk of extravasation and spread of infection and keeping the operative field clear. We have no instrumental complications or damage during the procedures.

## Limitations

Even though it is a prospective study, it has limitations. First, the number of cases enrolled in both groups (n = 33 in each group) was relatively small because the instances of partial staghorn calculi in our center were not numerous. Second, The second is that it is a single-center study. Also, it has a short-term follow-up, so it may need another long-term follow-up study to re-evaluate the possible remote postoperative complications.

## Conclusion

ECIRS is safe and effective in treating partial staghorn calculi either in the prone split-leg position or in the Galdakao-modified supine Valdivia position, with comparable outcomes and no statistically significant difference between both positions.

## Data Availability

The datasets used during this study are available from the corresponding author on reasonable request.
